# ‘I Was Shattered and Broken’: Unmasking the Experiences and Responses of Black Canadian to Pregnancy Loss

**DOI:** 10.1177/08445621251320570

**Published:** 2025-02-27

**Authors:** Priscilla N. Boakye, Nadia Prendergast, Ola Abanta Thomas Obewu, Diana Mugambi

**Affiliations:** 1 7984 Toronto Metropolitan University, Toronto, ON, Canada

**Keywords:** Black women, pregnancy loss, psychosocial burden, Canada, qualitative study

## Abstract

**Background:**

Pregnancy loss remains an invisible tragedy that impacts on the psychosocial well-being of women and their families. Cultural norms and beliefs about pregnancy loss affect how some women respond and process the loss. Yet research about Black Canadian women's experiences of pregnancy loss is lacking. The purpose of this research was to explore Black Canadian women's experiences and responses to pregnancy loss.

**Methods:**

A descriptive exploratory qualitative design was used to gain insight into the experiences of Black Canadian women. Semi-structured interviews were conducted with women who identified as Black. Data was analyzed using a thematic analysis approach.

**Results:**

We purposely recruited and interviewed 32 Black Canadian women who experienced miscarriage, stillbirth, or neonatal death. Three overarching themes were identified: (a) coming to terms with the reality of losing a pregnancy, (b) grappling with the psychosocial burden of losing a pregnancy, and (c) navigating for support after losing a pregnancy.

**Conclusion:**

Addressing the psychosocial burden of pregnancy loss is critical to promote the well-being of Black Canadian women. Nurses and other healthcare providers must recognize that the impact of pregnancy loss extends beyond the immediate clinical concerns. Therefore, intervention programs and follow up care must take a holistic and culturally responsive approach to address the needs of Black Canadian women beyond the period of the loss.

## Background

Pregnancy loss is a neglected public health tragedy that affects millions of women around the world. Globally, it is estimated that approximately 23 million of all pregnancies end in miscarriage (Quenby et al., 2021), and nearly 2 million stillbirths occur around the world yearly (United Nations Children's Fund [UNICEF], 2023). Although there is no data supporting the risk of stillbirth and miscarriage among Black women in Canada, the Public Health Agency of Canada (2020) has indicated that 15–25% of all pregnancies end in miscarriages. Evidence shows that there are inequities in stillborn rates within high-income countries, with those systematically marginalized being at greater risk (Flenady et al., 2016; UNICEF, 2020). For example, Black women in the United States are two times more likely to experience pregnancy loss (Ananth et al., 2022) while those in the United Kingdom have a 43% chance of having a miscarriage (Quenby et al., 2021) when compared to their White counterparts. Despite the increased risk of pregnancy loss among Black populations, research focusing on the experiences of Black women in Canada is lacking.

Beyond the life that is lost, women also grapple with the grief and psychosocial burden associated with pregnancy loss, which have far-reaching consequences on their everyday lives. Several authors have noted that pregnancy loss may intersect with racial and other social complexities to impede access to coping resources and complicate the grieving process of Black women (Antilla & Johnson, 2024; Evans et al., 2023; Fenstermacher & Hupcey, 2019). A recent study found that racism and discriminatory practices toward Black women continue to persist after pregnancy loss, including poor quality of care (Antilla & Johnson, 2024).

Pregnancy loss results in a cascade of emotional reactions that are often compounded by the lack of support and acknowledgement of the loss (Boyden et al., 2014; Jones et al., 2021). The limited understanding of the unique differences among affected women often results in the lack of acknowledgement and dismissal of their experiences (Bailey et al., 2019; Boyden et al., 2014; Evans et al., 2023). Further, because discussions surrounding pregnancy loss are often met with silence, many women may be unwilling to talk about their grief (Bellhouse et al., 2018; Pollock et al., 2021; World Health Organization [WHO], n.d.). Unacknowledged grief contributes to feelings of isolation, undermines coping, and hinders access to supportive care. Existing structural inequalities may limit access to comprehensive and culturally congruent therapy, which may exacerbate mental health vulnerabilities of affected women (Boakye et al., 2025).

Although pregnancy loss is a significant public health issue with profound social and mental health consequences, there is limited insight into the experiences of Black Canadian women. While a recent study explored the diverse experiences of women in Ontario, Canada (Watson et al., 2019), 87% of the sample were White, which may not account for the unique experiences of Black Canadian women. This paucity of research obscures the needs of Black Canadian women and hinders the development of programs to support them after pregnancy loss. Additionally, responses to pregnancy loss may be influenced by social and cultural norms regarding womanhood and childbirth. Therefore, understanding the ways Black Canadian women respond to, and cope with pregnancy loss is needed to inform the development of culturally relevant programs to address their needs. The purpose of this study was to explore the contextual influences on Black Canadian women's experiences and responses to pregnancy loss.

## Methods and Procedures

An exploratory descriptive qualitative design (Hunter et al., 2019) was used to gain in-depth insight into Black Canadian women's experiences and responses to pregnancy loss. The flexible and adaptive nature of exploratory descriptive qualitative design makes it appropriate for answering the research question guiding this study (Doyle et al., 2020; Kim et al., 2017). As an exploratory design, this approach allowed us to explore the experiences of the participants and to highlight the contextual influences on how they responded and coped with pregnancy loss.

### Sample and Recruitment

Thirty-two participants were recruited across the Greater Toronto Area. To be included in the study, the participant had to be 18 years and older, self identified as Black, and have experienced at least one type of pregnancy loss including miscarriage (<20 weeks), stillbirth (>20 weeks gestation) or neonatal death (newborn through 28 days of life). Recruitment fliers were distributed widely across Black community groups, women's groups, churches, and faith-based organizations. Participants who reached out to the research team completed an eligibility criteria form. Once the eligibility of a participant was confirmed, a secured Google form containing the consent form and a brief demographic questionnaire was sent to them to review and to decide whether they wished to participate in the interview. Given the nature of the topic, the interview guide included both unstructured and semi-structured questions to ensure participants had the opportunity to share their experiences in an empowering way. A copy of the interview guide has been attached as a Supplementary Material. Subsequent probing questions were used to explicate meaning from participants’ narration. The interviews were conducted via Zoom by PB and NP while OA and DM wrote down the field notes. Each interview lasted between 30–60 minutes and was digitally recorded. After the interviews were completed, each participant received a CAD $50 gift card to appreciate them for their courage and time to share their stories. Descriptive characteristics of the participants are presented in Table 1.

**Table 1.** Participants characteristics.

Demographic characteristics	N	%
Age		
18–23	0	0
24–34	26	81.2%
35–44	6	18.8%
Education		
High school	2	5.7%
College	8	25.7%
Bachelor's degree	15	40.0%
Postgraduate	7	25.7%
Religious affiliation		
No religion	3	9.4%
Christianity	26	81.25%
Islam	2	6.3%
Other	1	3.1%
Sexual orientation		
Heterosexual	30	93.7%
Queer	2	6.3%
Marital status		
Married	25	78.1&
Single	2	6.3%
Divorced	2	6.3%
Common-law	3	9.4%
Employment status		
Unemployed	3	9.4%%
Self employed	8	25.7%
Employed	21	65.6%
Income		
<$10,000	3	9.4%
$10,000–$19,999 CAD	3	9.4%
$20,000–$29,999 CAD	5	15.6%
$30,000–$39,999 CAD	4	12.5%
$40,000–$49,000 CAD	4	12.5%
>$50,000 CAD	13	40.6%
Type of loss		
Miscarriage (<20 weeks)	17	53.1%
Stillbirth (>20 weeks)	13	40.6
Neonatal death (loss before 28 weeks)	2	6.3%
Number of losses		
One loss	17	53.1%
2 or more	15	46.9%
Duration of loss		
Less than or equal to one year ago	21	65.6%
More than one year ago	11	34.4

### Data Analysis

We approached the analysis and interpretation using Braun and Clarke's (2021) thematic analysis approach. This method of analyzing qualitative data was chosen because it allows for a comprehensive understanding of participants experiences (Byrne, 2021; Squires, 2023). We began the initial stage of the analysis by carefully reviewing the audio recordings and written notes that were generated during the interviews. This stage of reviewing gave us preliminary insight into the data and helped us to probe more in depth during subsequent interviews. After the interviews were completed, we conducted a close reading and rereading of the transcripts to gain analytical insight and generated initial codes alongside. Two research team members, PB and NP, coded at least two transcripts each and all the research team met to review the initial coding and address any discrepancies. We revised the initial codes to enhance the consistency within our coding frame.

Subsequently, three of the authors, PB, OA and DM applied the codes to all the transcripts. We iteratively worked through the transcripts by highlighting the main ideas within the data and assigning codes to them. The codes were aggregated into tentative themes based on the common patterns and meanings emerging from the data. The tentative themes were reviewed by all research team members to ensure they were grounded in the data. After the themes were generated, we met frequently to review, define, and name the themes to capture their meaning and essence in relation to our research question. Once we reached a consensus on the themes, we generated a detailed and comprehensive summary of the themes and extracted relevant quotes from the data to illuminate the participants’ experiences. All quotes and extracts were carefully edited to improve readability without altering their meanings. We used NVivo to organize and manage the data analysis after all the transcripts were reviewed and edited for accuracy.

### Trustworthiness

In addition to conducting a robust analysis, we engaged in several strategies to enhance the rigour of our findings. We maintained rigour by showing consistency between our research questions and methodological approach. All research team members worked collectively to refine and finalize the emerging thematic ideas. Detailed field notes were kept during the interviews, and this was used to confirm and support our analytic impressions. We kept an audit trail of the analytic processes and revisited them to compare and ensure our analysis was informed by the participants’ data. We further engaged in reflexivity by recognizing how our positionality as Black nursing scholars and our preconceptions about pregnancy loss may impact the way we interpreted the data. To minimize these impacts, we frequently met via Zoom from the beginning of the analysis to the end to debrief and to ensure we achieved consensus in our interpretation.

### Ethical Considerations

The research received approval from Toronto Metropolitan University Research Ethics Board (REB 2023-374). All participants provided written informed consent that included a brief outline of the research purpose, expectations, risks, benefits, and rights of participants. Given the sensitive and distress-provoking nature of this research on our participants, we emailed a list of free counselling resources prior to the interview for participants to contact should they experience any distress after the interview. During the interviews, we frequently checked in on participants to ensure they felt comfortable proceeding with the interview. For participants who became emotional during the interview, we engaged them in deep breathing exercises and inquired if they wished to discontinue the interview. To protect the privacy of the participants, the transcripts were meticulously reviewed and all identifiable details that would link excerpts to participants were removed. The data were securely stored on a licensed Google Drive located at Toronto Metropolitan University.

## Results

Table 1 above presents the demographic information of the participants. More than half (51%) had a miscarriage, 40.6% experienced stillbirth, and 6.3% had a neonatal death. The majority of the participants (81.2%) were between the ages of 23–34 years and nearly 47% had two or more losses. Three overarching themes were generated to highlight Black Canadian women's experiences and responses to pregnancy loss. The themes and subthemes are discussed in the subsequent section below.

### Coming to Terms with the Reality of Losing a Pregnancy

#### Being in a state of numbness

Participants described experiencing a plethora of feelings and emotions after the pregnancy loss. Regardless of the type, pregnancy loss triggered a profound sense of disbelief and numbness as the reality of the loss became difficult for participants to bear. A participant described her initial reaction after witnessing the ‘cold and stiff’ body of her baby.

For the first few minutes after his birth, I felt completely numb. I couldn't cry, I couldn't do anything. The doctors and nurses gave him to me to look at him and to see what he looked like. And that was when it hit me how cold and stiff he was and that was when I started feeling the loss. At that point I was so numb. [Participant 13]

Others described their reaction as feeling “*immobilized”* [Participant 14], and another said, “*it is like my world stopped”* [Participant 20]. The sudden change from being an expectant mother to dealing with the loss of their baby left many struggling to comprehend the unfolding reality. A woman recounted: “*my mind went blank, and I couldn't comprehend. I really couldn't cry, and I remember standing somewhere and not really thinking anything and just staring still for two hours. I was just numb and not really believing”* [Participant 19]. These reactions enabled participants to temporarily disconnect themselves from the reality of the loss and the grief that was unfolding.

#### Denying the Experience of the Loss

Several participants described how they found it difficult to accept that they were losing or had lost their pregnancy. Symptoms of pregnancy loss, such as bleeding, were misconstrued by some participants as normal part of implantation process or deemed to be less serious. Such understanding offered a glimmer of hope, contributing to some women denying the impending loss. In recounting her experience of miscarriage, one participant narrated.

It was when the bleeding became too much that I went to the hospital. I did not want to go to the hospital. I just convinced myself that the baby is still there. Maybe the bleeding was just a normal implantation process. It was when the bleeding became serious then it dawned on me that I can no longer avoid it. [Participant 19]

For some of the women, denying that the loss was occurring enabled them to delay the grief and feelings of hopelessness that accompany pregnancy loss. Others who experienced stillbirth were convinced that their baby was asleep as stated by Participant 4: “*at some point, I tried to hit her to see if she would cry because I was like she wouldn't die. I’m very sure she would be sleeping or something*.” Because many stillborn infants’ bodily appearance do not show signs that indicate the absence of life, it led to a false sense of hope among some of the participants. Some participants indicated that pregnancy loss left them with a sense of illusion. This was captured by Participant 31 who stated: “*sometimes I will behave as if I am still carrying a baby but after some time, the thought will be coming back oh I've lost the pregnancy.”* Such mixed feelings created a false sense of hope and made it difficult for participants to accept the reality of the loss.

#### Going Into Isolation

Several participants described going into self-imposed isolation as they struggled to make sense of their grief. The deeply personal nature of the grief that accompanied pregnancy loss compelled some participants to withdraw and isolate themselves as they attempted to process the unfolding emotions. A participant shared her experience when she said: “*I was actually withdrawn and avoided being around people”* [Participant 12]. Other participants felt they went into isolation due to lack of understanding about the emotional pain that they were enduring at the time of their loss. A participant said:

I crave for my solitude more often. I wanted to be alone. I don't want to be with people. I tend to push people away because I feel like they don't really understand how I feel. I feel like I need more time alone to process everything. [Participant 20]

Participants felt that because “*people don't want to talk about it”* [Participant 24], coupled with the difficulty of having to painfully explain their loss to others without acknowledgment, caused them to isolate themselves. Some participants felt that the pregnancy loss affected their sense of belonging. This, along with the unrelatable nature of the emotions and grief that manifest after pregnancy loss, led them to withdraw from others.

I think experiencing loss is already such an isolating experience and then to feel just slightly not belonging further made me isolated. Because of the culture of not being able to talk about it and because your identity as an expectant mother has changed. It's hard because you can’t relate with anyone [Participant 14].

#### Living with the Blame

The loss of a pregnancy invoked a deep sense of responsibility and blame as several participants felt their actions or inactions contributed to the loss. As a result, participants became preoccupied with events and processes leading to the loss and asking questions as expressed in the following excerpt: *“what did I do wrong? where did I go wrong? what did I not do? So, I had a lot of questions going on in my mind after I lost my baby”* [Participant 8]. The need to accept blame triggered an intense feeling of guilt for not doing enough to save the life of their baby. One participant recounted:

I started blaming myself that there's something I didn't do right. You feel like there's something you should have done that would have prevented this and you just feel guilty because if I had done things in a certain way, it wouldn’t have happened. [Participant 17]

The perceived inability to successfully carry a pregnancy to term contributed to self-blame and led to feelings of hatred towards themselves. This further intensified participants’ belief that they were responsible for the loss.

I hated myself because I thought I did something wrong that actually made me lose my baby at that stage … I knew that deep down there was something I had done wrong or had not done right to make my baby stay with me. [Participant NO8]

Other participants stated that family members who found it difficult to accept the loss blamed them for not taking practical and timely decisions to avert the loss.

People try to put it on me for being the cause of everything and not taking swift action … I was made to feel that instead of me wallowing in grief. I should take the responsibility and the blame for things I've done wrong and why I am even in that situation. Everything really made me feel unloved. [Participant 20]

Such reactions from family members led to feelings of neglect and contributed to invalidation of the pain and grief experienced by participants.

### Grappling with the Psychosocial Burden of Losing a Pregnancy

#### Experiencing Shattered Expectations

The expectation of having a successful pregnancy was a much-anticipated outcome for many of the participants. Thus, pregnancy loss became an unexpected outcome that left participants feeling unprepared to adjust to the unfolding reality. Such a sudden change from being an expectant mother to grappling with a deep sense of loss made it difficult for participants to reconcile their feelings.

It is not easy to carry a baby for nine months and then after nine months, the baby is supposed to be like a kind of reward for your labour. And you know when you're supposed to get a reward, you discover that what you suffered for is gone. It really broke me, and it was shattering. [Participant 27]

For several of the participants, being pregnant was filled with many hopes and dreams of starting a family. The experience of pregnancy loss shattered these hopes and dreams and left them feeling disillusioned. In recounting her experience, a participant said, *“I felt so shattered … I had so much expectation and for a minute I was shattered even till today. No mother can really want to see and hold a child with no heartbeat”* [Participant 17]. Participants felt being pregnant was accompanied by an overwhelming sense of joy, but for many of them, this was suddenly replaced by grief and sorrow. Another participant re-echoing the sentiments of many others said: “*I thought pregnancy was supposed to bring happiness but to me it was different. I lost it and I was shattered and broken”* [Participant30]. Others who experienced multiple losses also felt pregnancy loss shattered the many plans they had envisioned.

I have so many hopes, high expectations I am going to give birth, and I am going to call my babies names, I am going to have fun with my child, play around, have my own small family, and then all of a sudden reality comes in, and then you now realize you're not going to experience this thing. [Participant 20]

#### Leaving with a Sense of Emptiness

Pregnancy created an emotional bond between participants and their developing fetus as they hoped to create a nurturing relationship after childbirth. Participants recounted how the experience of pregnancy loss severed this bond and overshadowed the joy that once permeated their lives. In summing the sentiments of many others, Participant 2 described the huge sense of void she felt after losing her pregnancy.

When you're pregnant, you start forming a bond with your baby. You're happy and you're prepared. But then again, you lose the baby. It's like you feel empty after having a miscarriage. So, it's like you've lost a part of you. It was a crucial part of you, and you lost it. So, you feel empty, and it is like something is missing. [Participant 2]

The irreversible nature of the loss and the thought of not being able to immediately replace the developing fetus exacerbated the feelings of participants that something of theirs was missing. A participant said: “*I still feel something is missing. I feel that something is missing. I don't feel complete after the loss of the baby. I feel like I've lost something precious to me”* [Participant 7]. Pregnancy loss also disrupted an identity that was once embraced by the participants as they envisioned their role of becoming mothers.

Losing my baby means a lot. It is as if a part of my body left me. I have actually had miscarriages in the past. So, I kept on trying and I was finally going to have a child, and she could not leave, she could not actually enjoy life. So, losing her is as if a part of me naturally left me. [Participant 12]

Such feelings of having part of their identity taken away served as a constant reminder of the loss, leaving participants grappling with prolonged emotions of incompleteness and a pervasive sense of emptiness.

#### Feeling of Being a Failure

The experience of pregnancy loss was accompanied by a perceived sense of personal failure and inadequacy. Participants perceived pregnancy and childbirth as a fundamental part of their womanhood and measure of their social standing. Many felt that experiencing pregnancy loss set them apart from their peers and led to the perception that they were incapable of carrying a pregnancy to term. This contrast between them and their peers who successfully gave birth became a constant reminder of their inability to fulfill their role.

I felt I embarked on a journey, and I failed. I failed woefully … Most of the things that made me feel like I am a failure was during that time; I had a friend of mine who was also pregnant and she didn't lose her child. The fact that she's carrying a child, and I have lost mine … Things like this made me feel like I'm a failure and also contributed to me thinking that I have failed. It's like I failed an exam, an exam that other people usually write and pass, but I failed. [Participant 2]

Given the high value placed on carrying a pregnancy to term, losing a pregnancy fueled a feeling of bodily betrayal and contibuted to their perceived sense of failure. One participant stated: *“I felt like my body wasn't capable of carrying a baby. So, I felt like there was a rejection of the baby from my body which made me feel like a failure*” [Participant 24]*.* Some participants also felt their inability to fulfill their perceived roles as women and meet the expectations of their partners and family members intensified their feelings of being a failure.

At some point I did see myself as a failure because everybody was very excited about my pregnancy, and I was treated very well. So, I just saw myself as a failure because I failed them. It felt like I had failed my husband and his family. [Participant 3]

The perceived sense of failure was accompanied by an overwhelming feeling of shame for letting down their partner and family as captured by one participant who said: “*I couldn't even look at my husband in the face. I was ashamed. I felt ashamed. I felt like society might mock people like me”* [Participant 22]. Like other participants, the fear of being mocked and judged by family and friends for failing to meet expectations intensified their shame.

#### Feeling of not Belonging

Participants described losing a pregnancy affected their sense of womanhood which in turn made it difficult for them to feel accepted by others. The participants were aware of their role as women was closely intertwine with their ability to bear children. This understanding was expressed by Participant 2 when she stated: “*our value as Black women is associated with our ability to reproduce. I think it's also generational … that is how we identify our value as women”* [Participant 2]. For some participants, pregnancy loss resulted in their inability to prove their reproductive capacity, leading to diminished self-worth. In sharing her experience, Participant 24 stated that losing a pregnancy “*felt like I was incapable of being a mother or carrying a child … I was sort of less than other people who were able to carry babies to term.”* The expectation to marry and have children was not only a personal desire of the participants but one that was shaped by societal beliefs about their reproductive capacity. Some participants felt that being unable to successfully carry a pregnancy to term affected their sense of accomplishment and purpose.

I lost a sense of belonging, I lost sense of direction, I felt I could never be a mother, I felt like I disappointed my husband, I felt like I was not a woman, I felt like I lost the joy of being a woman. The joy of being a woman was lost, and I felt a part of me was taken away from me … I didn't belong in my marriage. I felt I didn't belong to my husband. I felt like I had lost the purpose of living, and I have really not accomplished anything despite my personal achievements. [Participant 19]

For some participants who perceived pregnancy as a default pathway to motherhood and had a strong desire to become mothers, losing a pregnancy was considered a missed opportunity. Participant 7 stated that losing a pregnancy “*means a lot to me and that I wouldn't be a new mom. I lost the privilege of being a mother and it felt like I lost what I really desired in life to become a mom.”* Other participants indicated that losing the privilege of becoming a mother “*felt like my marriage was gonna crumble if I was not able to have a child”* [Participant 18]. The inability to bear children provoked feelings of marital insecurity in some participants.

#### Facing the Uncertainty About Future

Pregnancy loss invoked an intense fear that created a shift in participants’ perceptions and decisions about future pregnancies. The loss of pregnancy made it difficult for participants to reconcile their present experiences and the hopes and dreams they had envisioned. A participant narrated how experiencing both miscarriage and stillbirth changed her perspective and made her to reconsider her plans of getting pregnant again.

At some point, I was like let me just take a break from childbearing. The stillbirth and the miscarriage had a very big impact on my life … I told my husband that I need to take a break. I don’t want to go through pregnancy at the moment … I feel so scared that if I go through that path again, I may have a miscarriage or have another stillbirth or lose my life … so I have put everything on hold now. [Participant 10]

The aftermath of dealing with a pregnancy loss was a dreadful experience that left many participants grappling with the question of ‘what if it happens again’. This fear shared by several other participants was captured in the following excerpt by Participant 19 who said: “*I was really scared of getting pregnant again because I don't know what I will do if I lose another child.”* Pregnancy loss left behind a lingering fear resulting in some participants feeling anxious about the prospects of future pregnancy. This perception was expressed by Participant 8 who stated: *“each time I imagine that I have to get pregnant, I have this anxiety and panic attack … about what to do next when I get pregnant, and what not to do.”* The inability of some participants to overcome this fear also affected their sexual life as many struggled to maintain intimacy with their partners. A participant said: “*before the loss we were always trying for a child and I was deliberately having sex in order to get pregnant, but after the child lost, I just decided it was best if we stopped ‘*’ [Participant 32]. The joy of getting pregnant and having children that once dominated their lives was now clouded by a pervasive sense of fear and uncertainty.

### Navigating for Support After Losing a Pregnancy

#### Coping with Inadequate Professional Support

Participants described an overwhelming lack of comprehensive support to deal with the emotional turmoil they experienced after a pregnancy loss. In instances where support was provided, some participants felt it was fragmented and inadequate. A participant shared her experience when she was asked about what support was made available to her: *“they just asked me am I okay, am I feeling okay? There was no sort of emotional support”* [Participant 1]. Another participant also described being left to go home without any effort to provide her with the necessary support.

I mean like there was nothing. The only thing I remember them doing was handing me my baby to see before and making a decision how I wanted him to be laid to rest. We left the hospital after that, and I wasn't offered anything … if I were white, it would be different, probably that's how our system works. [Participant 13]

Similar sentiment was re-echoed by Participant 29 who stated: “the [nurses and healthcare providers] really were not concerned about my mental health and the emotions that I had during or immediately after the loss.” In the absence of support, several participants sought the services of a therapist to help them cope with the grief and the mental health struggles they experienced after the loss.

I knew that I was not really okay mentally, so I had to hire a personal therapist for myself because I was losing it. I had images of what my baby looked like everywhere. I knew I needed help, so I got a personal therapist. [Participant 20]

While some participants alluded that they were provided with a therapist from the hospital, many felt the services were not culturally concordant resulting in Participant 29 opting out for a Black therapist.

They were willing to give me some therapy sessions, but I think I prefer going for therapy in the institution that I'm currently going to … because the therapist is Black … And I think this helps with the effectiveness of the therapy sessions. [Participant 29]

Some participants described the financial burden of paying for these services limited them from fully accessing and receiving the support they needed to deal with the emotional challenges.

I still have to go for therapy … I feel the financial drain is too much and so overwhelming. I think it will be the best if it was provided or there was funding that would help. It's really stressful because this is not something that you planned for, so it's just a burden you have to take just because you want to heal. So, it is financially draining. [Participant 15]

Although participants considered going for therapy to be key to their recovery, the unexpected burden of cost associated with paying for the services had a significant toll on their finances. Several participants suggested the need for funding programs to help offset the cost of therapy sessions, and until that happens it is a “*double loss because I lose the baby and again lose finances to treat my mental health.”* [Participant 29]

#### Thriving Through Faith and Family

In the absence of comprehensive institutional support systems, the participants relied on their faith and personal coping resources. Through prayers and spiritual appraisal, participants were able to repurpose their grief and healing. A participant described how leaning into her faith gave her the strength and hope to move forward.

I prayed so much. Because as a Christian, we believe that there is nothing that God cannot do. And even as I cried and those tears flowed down, I felt relieved as I listened to music, it gives me hope. I don't mean any kind of music; I mean music that helps you build your faith in God. Music that reminds you that God is still thinking about you. I prayed and I believe God is going to come through for us. [Participant 20]

Others described drawing on positive self-affirmation to overcome their grief as captured in the following excerpt: “I said positive things to myself like I can make it, I can pull through … I was able to talk to myself with those positive words, motivational words to pull through” [Participant 26]. By staying positive, participants were able to put their loss into perspective and practice self-compassion.

I practiced self-compassion and gratitude, which actually helped me out … Every day I wake up, I look into myself, I look into the mirror, looking at myself, and I talk strongly with myself, that I am definitely going to get through my tough moments. [Participant 8]

Through self-compassion, participants found ways to cope with the loss and avoided judging themselves harshly. Some also described how they coped with the loss by redirecting their efforts towards supporting others going through similar situations.

I felt like I wanted to get more involved … I became a volunteer, and I offered one on one phone support, which I received also as the grieving parent, and I also helped facilitate online groups. And this was a way that I really felt like I could connect with other families and share my experience. [Participant 24]

By sharing their experiences with others, participants found meaning and purpose as they translated their pain into hope. Others described that the support they received from their partners was instrumental in providing them with the comfort they needed.

My husband did so many things to support me … Everything meant a lot because I didn't feel insecure. He never made me feel insecure. He never made me feel like I was less of a woman. It made me feel like he didn't judge me. Sometimes a lot of marriages have issues simply because of miscarriage … so I also thought it was going to happen to me, but he gave me reasons not to. So, my husband was my strongest support system. [Participant 22]

For some participants, their partners being a “*main pillar of strength”* [Participant 25] allayed their fears and provided them with the assurance that they were not alone. Others also described receiving emotional support from their family members as expressed by one participant who said: “*I received support from family members and my mother who supported me emotionally”* [Participant 11].

#### Finding Solace Through Online Resources

Participants found solace by connecting with others through online groups. One participant stated: “*I sought solace in some support groups and online communities to actually heal … It actually helped me to heal part of the pain I was feeling”* [Participant 8]. Several others also described that joining online groups provided them with a sense of community and the support they needed in their moment of grief and pain.

I join groups online. I met with people that have experienced the same thing, and they talked about how they actually coped. They encouraged me that it was not the end of my life, that you could still make babies after a loss. I use that in encouraging myself. [Participant 12]

Some participants felt that while online platforms offered them the opportunity to have their feelings validated and acknowledged, they also described an overwhelming lack of representation among those facilitating the online group session.

There is an Instagram group that I joined but it was mostly run by Whites. Within the Black community there was nothing, so I had to create a group of women who also had gone through miscarriage or stillbirth. I organized them and we met once a week to discuss how to cope with things and how to help each other grow and come out of it. So those really strengthened me and made me feel seen, heard, and loved during my difficult times. [Participant 13]

The absence of a Black-focused online support group, while not surprising, highlights the struggles many go through navigating for support. Finding the space to share and be heard by people who reflected them compelled Participant 13 to create a Black-focused group that provided her and others the safe space to collectively heal together.

## Discussion

Our findings revealed that socio-cultural norms and expectations about pregnancy and childbirth influenced how Black Canadian women made sense of their experiences following a pregnancy loss. Yet the care of Black Canadian women after pregnancy loss has focused much on treating the clinical aspects of the loss to the complete neglect of how these socio-cultural influences complicate their healing and recovery process. WHO (n.d.) has indicated that failure to recognize the sociocultural influences and psychosocial burden associated with pregnancy loss is a major contributory factor to the overall lack of care and programs to support affected women and their families. The findings of our study highlight the need to de-medicalize the care of women after pregnancy loss, and call to attention the importance of taking a broader and comprehensive approach to address the needs of Black Canadian women.

Black Canadian women's reaction to pregnancy loss affected how they processed the grief that ensued. Studies have shown that while such reactions to pregnancy loss may serve as a protective shield against the immediate impact of losing a baby (Bailey et al., 2019; Kelley & Trinidad, 2012), it also reveals the difficulty many women live with reconciling their expectations and the reality of losing a baby. Despite the causes of pregnancy loss being inevitable and out of women's control, Black Canadian women were blamed by their families for not taking immediate action to avert the loss. Such experiences contributed to invalidation and lack of acknowledgement of their loss and grief, making it challenging for Black Canadian women to accept and heal from the loss. Consistent with other studies, Black women unjustifiably accepted responsibility for their perceived inactions for being unable to prevent the loss (Evans et al., 2023; Jones et al., 2021; Kavanaugh & Hershberger, 2005; Kelley & Trinidad, 2012). The tendency of women to unjustifiably blame themselves was reinforced by societal expectations about the role women play in protecting the life of the unborn baby (Kłos-Skrzypczak, 2023; Omar et al., 2019; WHO, n.d.). With no outlet for discussing their feelings, Black Canadian women became obsessively occupied with the thought of not doing enough to prevent the loss.

Because discussions about pregnancy loss are often met with silence in some cultures, women may be unwilling to talk about their grief (Bellhouse et al., 2018; Pollock et al., 2021; WHO, n.d.). Similar to the findings of this current study, several others have reported that discussions about pregnancy loss are often avoided within Black communities, leading to silence and suppression of the feelings among affected women (Antilla & Johnson, 2024; Evans et al., 2023; Jones et al., 2021; Kavanaugh & Hershberger, 2005). The perception that losing a pregnancy or child is a bad omen or taboo results in the stigmatization of women and hinders them from openly discussing their traumas (Pollock et al., 2020; UNICEF, 2020).

In communities where motherhood is valued and celebrated, women may feel alienated and disconnected from their partners, families, and communities (WHO, n.d.). Pregnancy confers a sense of identity, status, and belonging within one's community. The findings of our study revealed that the experience of pregnancy loss resulted in the disruption of affected women's identity. Prior studies have reported that pregnancy loss triggers an identity crisis as women struggle to make sense of themselves (Fairchild & Arrington, 2022; Minton et al., 2023). This, along with fear and uncertainty about the outcome of future pregnancies contributed to many participants in our study having difficulty establishing sexual intimacy and emotionally distancing themselves from their partners.

Black Canadian women in our study reported the lack of concern from nurses and healthcare providers regarding their mental health after pregnancy loss. Failure to prioritize the mental health needs of Black women has been reported to undermine their journey toward healing (Hill, 2019; Van & Meleis, 2003). Access to mental health support offers affected women the space where their loss and grief can be validated and acknowledged. However, barriers related to affordable access to culturally informed therapy sessions in the aftermath of the loss were lacking for the participants in this current study. As a result, Black Canadian women engaged in multiple coping strategies by drawing on their faith, internal coping resources, and family network. Similar strategies have been reported in previous studies where spirituality and family support systems were common coping strategies used by Black women after pregnancy loss (Antilla & Johnson, 2024; Jones et al., 2021; Van & Meleis, 2003). Although the women in our study found online support platforms provided them with a sense of belonging, comfort, empathy, and understanding, our findings also highlighted the lack of diversity, representation, and culturally responsive online support groups.

## Recommendations

Holistic, comprehensive, and integrated programs to support Black Canadian women experiencing pregnancy loss are urgently needed to address the psychosocial burden faced by many. This will require prioritizing equitable access to professional mental health support. Such change will contribute to minimizing the financial burden placed on families who often pay for professional therapy sessions. Alongside this, it is imperative to ensure that support programs must be culturally informed and tailored to meet the needs of Black Canadian women.

Addressing the emotional needs of Black Canadian women who experience pregnancy loss, both in the immediate and aftermath is critical to promoting their mental well-being. The proximity of nurses at the bedside provides an opportunity to respond to the emotional needs of affected women and to also facilitate access to resources and services. Such support must go beyond handing over leaflets and books. It should involve directly connecting women to counselling resources and support groups. Much of the challenges women face occur after they are discharged home. Thus, nurses and healthcare providers need to be educated on the sociocultural factors that shape the ways Black Canadian women process and deal with the aftermath of pregnancy loss. It is therefore imperative for nurses to have scheduled follow-up care to ensure that women who experience pregnancy loss are assessed and screened for any potential mental health and other socio-cultural issues that might impact on their healing and recovery. Normalizing discussions around pregnancy loss through creating awareness within Black communities and breaking the culture of silence is a critical step towards healing. This will ensure spaces are created to empower women to openly speak about their loss and feel supported.

## Strengths and Limitations

While there may be other Canadian studies which included the experiences of Black Canadian women, our study offers more nuance and contextual understanding on the experiences and responses of Black Canadian women to pregnancy loss. We also recognize that our study is not without limitations. First, the study recruited participants within the Greater Toronto Area and may not represent the experiences of women in other Black Canadian women in other provinces. Second, access to care and support after pregnancy loss may vary across Canadian provinces. While the findings of our study may not be generalizable, they may be transferable to other Canadian provinces. Given the significant dearth of research on Black Canadian women's experiences of pregnancy loss, further research is warranted to examine the prevalence of pregnancy loss among this population. In addition, research is also needed to analyse the current models of care and support programs to determine if they address the sociocultural challenges experienced by Black Canadian women after pregnancy loss.

## Conclusion

Our study sheds light on a tragic yet neglected issue in nursing and healthcare discourse that has profound consequences for the social and mental well-being of affected women. The findings serve as a call to action to improve access to equitable support and to create awareness to normalize the experience of pregnancy loss.

## Supplemental Material

sj-docx-1-cjn-10.1177_08445621251320570 - Supplemental material for ‘I Was Shattered and Broken’: Unmasking the Experiences and Responses of Black Canadian to Pregnancy Loss Supplemental material, sj-docx-1-cjn-10.1177_08445621251320570 for ‘I Was Shattered and Broken’: Unmasking the Experiences and Responses of Black Canadian to Pregnancy Loss by Priscilla N. Boakye, Nadia Prendergast, Ola Abanta Thomas Obewu and Diana Mugambi in Canadian Journal of Nursing Research
