# Supplementary material for: ‘I Was Shattered and Broken’: Unmasking the Experiences and Responses of Black Canadian to Pregnancy Loss
Source: Can J Nurs Res. 2025 Feb 27;57(3):341–51. doi: 10.1177/08445621251320570 (PMC12344207; doi:10.1177/08445621251320570)
Supplement: sj-docx-1-cjn-10.1177_08445621251320570 - Supplemental material for ‘I Was Shattered and Broken’: Unmasking the Experiences and Responses of Black Canadian to Pregnancy Loss [file sj-docx-1-cjn-10.1177_08445621251320570.docx]

**Interview Guide**

Introduction: Thank you for agreeing to take part in this study. We are interested in learning more about your experience after a pregnancy loss, and what are your needs.

1. Please tell me about your experience of having a pregnancy loss or the death of your newborn baby.
2. What does the loss of your pregnancy or newborn baby mean to you?
3. How did you feel after the loss of your pregnancy or newborn baby
4. What factors at home, in the healthcare system, and in the community facilitated or impeded your coping following the pregnancy loss?
5. Many people who experience pregnancy loss find community services and resources helpful in supporting their coping. Based on your awareness, what mental health support, services and resources are available to you.
6. What recommendations do you have about programs and services to promote the wellbeing of Black women who experience pregnancy loss?
7. What else, related to the pregnancy loss would you like to add that we have not discussed?
